# Exploring immune status in peripheral blood and tumor tissue in association with survival in patients with multi-organ metastatic colorectal cancer

**DOI:** 10.1080/2162402X.2024.2361971

**Published:** 2024-06-10

**Authors:** Lotte Bakkerus, Beatriz Subtil, Hetty J. Bontkes, Elske C. Gootjes, Martine Reijm, Manon Vullings, Kiek Verrijp, John-Melle Bokhorst, Carmen Woortman, Iris D. Nagtegaal, Marianne A. Jonker, Hans J. van der Vliet, Cornelis Verhoef, Mark A.J. Gorris, I. Jolanda M. de Vries, Tanja D. de Gruijl, Henk M.W. Verheul, Tineke E. Buffart, Daniele V. F. Tauriello

**Affiliations:** a Department of Medical Oncology, Radboud University Medical Center, Nijmegen, The Netherlands; b Department of Medical BioSciences, Radboud University Medical Center, Nijmegen, The Netherlands; c Department Laboratory Medicine, LGDO, Section Medical Immunology, Amsterdam, The Netherlands; d Department of Pathology, Radboud University Medical Center, Nijmegen, The Netherlands; e Department of IQ Health, Radboud University Medical Center, Nijmegen, The Netherlands; f Department of Medical Oncology, Cancer Center Amsterdam, Amsterdam UMC, Location VUmc, Amsterdam, The Netherlands; g Department of Surgery, ErasmusMC Cancer Institute, University Medical Center Rotterdam, Rotterdam, The Netherlands; h Department of Medical Oncology, Erasmus MC Cancer Institute, University Medical Center Rotterdam, The Netherlands

**Keywords:** Clinical-stage research, colorectal cancer, metastasis, prognostic biomarkers, systemic immunity, tumour immune microenvironment, tumour immunity

## Abstract

Colorectal cancer (CRC) raises considerable clinical challenges, including a high mortality rate once the tumor spreads to distant sites. At this advanced stage, more accurate prediction of prognosis and treatment outcome is urgently needed. The role of cancer immunity in metastatic CRC (mCRC) is poorly understood. Here, we explore cellular immune cell status in patients with multi-organ mCRC. We analyzed T cell infiltration in primary tumor sections, surveyed the lymphocytic landscape of liver metastases, and assessed circulating mononuclear immune cells. Besides asking whether immune cells are associated with survival at this stage of the disease, we investigated correlations between the different tissue types; as this could indicate a dominant immune phenotype. Taken together, our analyses corroborate previous observations that higher levels of CD8+ T lymphocytes link to better survival outcomes. Our findings therefore extend evidence from earlier stages of CRC to indicate an important role for cancer immunity in disease control even after metastatic spreading to multiple organs. This finding may help to improve predicting outcome of patients with mCRC and suggests a future role for immunotherapeutic strategies.

## Introduction

Colorectal cancer (CRC) is the second leading cause of cancer-related deaths worldwide.^1^ The standard treatment for patients with extensively metastatic CRC (mCRC) involving mismatch repair proficient/microsatellite stable (pMMR/MSS) tumors is systemic therapy with chemotherapeutic and biological agents. The median overall survival (mOS) of patients who received adequate systemic treatment is currently approaching 3 years. Patients with right-sided, *BRAF*-mutant cancers generally have the lowest survival.^2,3^ However, treatment response and clinical outcome of patients vary greatly within these subtypes. In the absence of precise biomarkers, patients are exposed to toxic treatments that may strongly deteriorate their quality of life, yet do not offer survival benefit for non-responders. A better biological understanding of the metastatic process may inspire the development of accurate biomarkers as well as new therapeutic strategies.^4,5^

Cancer immunity may have a pivotal role in this understanding. In stage I – III CRC, it was shown that the host adaptive immune reaction and T cell infiltration in the primary tumor correlate to less disease recurrence and better OS.^6,7^ By spreading to distant organs, mCRC has escaped several levels of immunosurveillance.^8^ There is mounting evidence suggesting an important role for cancer immunity in metastatic onset,^9,10^ as well as for therapeutic potential for immune checkpoint blockade (ICB) in combination with overcoming the suppressive tumor microenvironment (TME) in pMRR/MSS mCRC in preclinical models.^11,12^ Nevertheless, only patients with MMR-deficient/microsatellite instable (dMMR/MSI) tumors benefit from ICB.^13^ Studies analyzing the TME of metastases in patients with resectable disease found that high T cell infiltration is also associated with improved survival outcomes there.^14–22^ Immunological variables and their relationship to survival have not been studied in patients with multi-organ mCRC.

Both the local immune landscape and the systemic immune response are relevant.^23–26^ The role of circulating immune cells as a potential biomarker is of interest in both early-stage and advanced CRC, relating to metastasis initiation as well as growth dynamics. The association between immune variables in peripheral blood and clinical outcome in patients with mCRC, or between systemic immune status and tumor tissue infiltration, has been investigated.^27–31^ These studies indicate prognostic power of readily accessible systemic factors such as cytotoxic CD8+ or regulatory T (Treg) cell counts. However, research has also identified a complex and plastic relationship between tissue and the immune macroenvironment.^25,32^

The primary objective of our exploratory study was to assess immune profiles in both peripheral blood and the local TME as potential biomarkers for survival in patients with systemically spread mCRC before the start of first-line palliative systemic therapy. Although this setting may be expected to feature a defunct immune status, we hypothesize that evidence for local/systemic anti-cancer immunity may yet correlate with better survival even in these late stages of the disease.

## Materials and methods

### Patients

The ORCHESTRA trial is a randomized multicenter clinical trial for patients with multi-organ mCRC, comparing the combination of chemotherapy and maximal tumor debulking versus chemotherapy alone (NCT01792934). Patients were 18 years or older and had an indication for first-line palliative systemic therapy for mCRC. Comprehensive in- and exclusion criteria are available at clinicaltrials.gov. Patients included in the present study were treated with capecitabine and oxaliplatin (oxaliplatin IV followed by 14 days of oral capecitabine in a 3-week cycle; CAPOX) with or without bevacizumab. A CT scan of thorax and abdomen was performed after 3 cycles. Follow-up scans were performed at least every 3 months until disease progression. Patient inclusion of the trial has been completed in September 2023. The side-studies in subgroups of the included patients, as described here, were performed and analyzed without knowledge of the final outcome of the two study arms in the ORCHESTRA clinical trial. We determined available follow-up data as of July 2023: dates of progression event/death were used to compute PFS/OS in our analyses. OS was defined as the time period from the day of inclusion until death from any cause. PFS was defined as the time period from the day of inclusion until progressive disease according to Response Evaluation criteria in Solid Tumors (RECIST version 1.1). None of the clinical cohorts contained censored survival observations.

Clinical data included age, sex, primary tumor sidedness (right, left, or rectum), primary tumor *in situ* (yes or no; y/n), primary tumor differentiation (well/moderately or poor), number of organs involved in metastatic disease (2 or >2), chronicity (synchronous or metachronous), prior (neo)adjuvant treatment of the primary tumor (y/n), number of metastatic lesions (<5, 5–10, >10, or diffuse peritoneal disease), metastatic location (including only the most common metastatic sites: liver y/n, lung y/n, lymph node y/n, or peritoneum y/n), prior local treatment of metastases (y/n), treatment with bevacizumab (y/n), CEA and LDH levels at baseline (normal or elevated), *BRAF*/*KRAS*/*NRAS* mutation status (wildtype or mutated), MSI status (MSS *vs* MSI), see Table S1.

**Figure 1.**
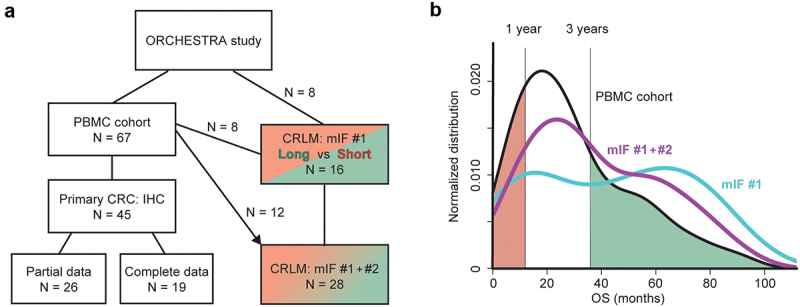
Overview of the study sub-cohorts and their general survival distribution.

Blood samples were prospectively collected as part of the preplanned translational study program of the trial from May 2013 to October 2017. Of in total 70 patients, peripheral blood mononuclear cells (PBMC), baseline metastasis needle biopsies, and formalin-fixed and paraffin-embedded (FFPE) primary CRC tissue samples were collected. Three patients were lost to follow up due to withdrawal of consent. Immunohistochemistry (IHC) analysis was performed on 45 samples of primary CRC included in this cohort (Figure 1a). In addition, a group of 16 ORCHESTRA patients with available FFPE material of liver metastases with having either a short (<1 year) or long (>3 year) survival were selected for tissue analyses. Since 9 of these were not represented in the original PBMC group, we added 12 additional samples from the PBMC cohort (not selecting for survival; Figure 1a). Multiplex immunofluorescence (mIF) analysis was performed on the combined dataset, batches mIF#1+#2. Patient follow-up data were retrieved in July 2023. Written informed consent was obtained from all patients included in the ORCHESTRA trial. The study protocol was reviewed and approved by the institutional review board of the Amsterdam UMC and the study was performed in accordance with the Declaration of Helsinki. Clinical data are summarized in Table S1.

(a) Schematic of the sub-cohorts used. *PBMC*: peripheral blood mononuclear cell assessment (flow cytometry); IHC: immunohistochemistry of primary CRC sections (the fraction with all 4 variables is indicated, *complete* vs *partial data*); *mIF*: multiplex immunofluorescence on CRC liver metastases (*CRLM*). (b) Distribution of OS of the mIF batches (#1, blue, and the combination of #1 and #2, purple) in relation to the PBMC cohort. Populations with short/long survival are indicated (red/green).

### Flow cytometry PBMC analysis

Blood samples were collected at baseline, within 7 days prior to the start of systemic therapy. Samples were collected in EDTA tubes and analyzed as previously described.^33^ Samples were shipped to central laboratory at Cancer Center Amsterdam, Amsterdam UMC and processed within 48 hours. T cell subset analysis was performed on pre-washed whole blood, to prevent sequestering of CD25 antibody by plasma CD25, gated on the PBMC population by CD45 expression and sidward scatter as shown in Figure S1. Acquisition was done with a 3-laser Gallios flowcytometer and data were analyzed with Kaluza (both Beckman Coulter). The following monoclonal antibodies were used for phenotyping of lymphocytes and monocytes: CD19-ECD (clone J3–119), CD45RA-ECD (2H4), CD27-PC7 (1A4CD27), CD56-PC7 (N901), CD8-APC-AF700 (B9.11), CD45-KO (J33) (Beckman Coulter); IgG1-FITC (X40), CD14-PerCP (MφP9), CD3-APC (SK7), CD25-PE (2A3), CD4-APC-H7 (SK3) (BD Biosciences); PD1-FITC (MIH4) (BD Pharmingen), and HLA-DR-V450 (L243) (BD Horizon). For analyses of absolute and relative numbers of CD14+ monocytes, CD19+ B cells, CD3+ T cells, and CD56+CD3- natural killer (NK) cells, 50 μl of whole-blood was stained in Trucount Tubes (BD Biosciences) using a lyse-no-wash procedure, and absolute cell counts per μl blood were calculated according to manufacturer’s instructions. After staining, cells were fixed and erythrocytes were lysed with Optilyse B (Beckman Coulter) according to manufacturer’s instructions.

The lymphocyte and monocyte subsets were defined as follows: CD19+ B cells, CD3-CD56+ NK cells, CD14+ monocytes (although CD14+ dendritic cells cannot be distinguished), and HLA-DR low/negative CD14+ monocytic myeloid-derived suppressor cells (MDSC). CD3+ T cells were divided into CD4+ and CD8+ lineages. CD4+ T cells were classified into (CD4+) CD45RA+ naive (CD4_N_) cells, CD45RA- memory (CD4_M_) cells, and CD25^hi^ regulatory T (Treg) cells^34^—further divided into (CD4+ CD25^hi^) CD45RA- active Treg cells and CD45RA+ resting Treg cells. For the CD8+ lineage, we defined (CD8+) CD45RA+CD27+ naïve (CD8_N_) cells, CD45RA-CD27+ central memory (CD8_CM_) cells, CD45RA-CD27- effector memory (CD8_EM_) cells, and CD45RA+CD27- effector cells (terminally differentiated EM cells re-expressing CD45RA, CD8_EMRA_). Furthermore, CD25, PD1, and HLA-DR expression on T cell subsets was analyzed. Relative values are a percentage of parent gate. See Supplementary Fig. S1 for gating strategies.

### Immunohistochemistry analysis on primary CRC samples

From FFPE tissue blocks of primary tumors, sequential slides were stained with H&E and with anti-CD3 and anti-CD8 (DAKO Omnis GA503 Rabbit polyclonal, and GA623 clone C8/144B). Slides were digitized using the Pannoramic P1000 (3D-Histech, Budapest, Hungary) at 40X magnification (0.24 × 0.24 μm/pixel). On each of the CD3/CD8 slides, regions of interest (ROIs) of 0.8 × 0.8 mm at the tumor center as well as the invasive margin were manually annotated by an expert (CW) to highlight regions with dense T cell infiltration, in line with evidence that this methodology of hot spot selection yields better results than random region selection.^35^ These annotations were used to apply a deep-learning technique and compute the number of lymphocytes in the selected regions as previously described.^36^

### Multiplex immunofluorescence (mIF) analysis on liver metastases

FFPE samples of CRC liver metastases were sectioned into 4 µm-thick slices and stained as described.^37^ mIF stainings were performed using the BOND-Rx Fully Automated IHC and ISH system (Leica Biosystems) with an Opal 7-Color Automation IHC Kit (NEL821001KT, Akoya Biosciences). Specifications for antibodies and Opal dyes were: anti-pan-cytokeratin (AE1/AE3 + 5D3; Abcam, ab86734; 1:1500; Opal650), anti-CD3 (SP7; Thermo Fisher; 1:200; Opal520), anti-CD8 (C8/144B; Dako, M7103; 1:200; Opal690), anti-FOXP3 (236A/E7; eBioscience, 14–4777; 1:100; Opal540), anti-CD56 (MRQ-42; Cell Marque, 156 R–94; 1:500; Opal620), anti-CD20 (L26; Thermo Fisher, MS-340; 1:600; Opal570). The slides were counterstained with DAPI for 5 minutes and enclosed in Fluoromount-G mounting medium (SouthernBiotech, 0100–01). Tissue slides were scanned at 4× magnification using the microscope Vectra 3 Automated Quantitative Pathology Imaging System (v3.0.4, PerkinElmer). ROIs, containing both tumor and surrounding stroma, were drawn with the aid of the corresponding H&E stainings using the PerkinElmer Phenochart software (v1.0.9). For further analysis, ROI were imaged at 20× magnification. InForm image-analysis software (v2.4.2, PerkinElmer) was used for spectral unmixing of Opal fluorophores, removal of autofluorescence signal, and tissue segmentation.

For segmentation (epithelial tumor fields *versus* stroma), an algorithm was trained based on the expression of pan-cytokeratin, DAPI, and autofluorescence. Subsequently, cell identification, segmentation, and phenotyping were performed by an in-house developed neural network (ImmuNet).^38^ The resulting data were exported in Flow Cytometry Standard (FCS) files, and cell populations were gated and quantified in FlowJo (version 10, Tree Star Inc., Ashland, OR, USA). Infiltration of immune cells was expressed in cell density by dividing absolute cell counts by surface area (mm^2^) of the tissue region (intraepithelial or stroma).

### Statistical analysis

For univariable analyses, Cox proportional hazards models were used (in SPSS v29 or in R/R studio v4.3.0/v2023.06.1 using the *survival* package v3.5–5) to estimate the association of continuous variables with survival – scaled to an increment of 100 cells or 10%-points to compute hazard ratios (HR). For categorical comparisons, patients were split into 2 (median) or 3 (tertiles) groups; or in four groups based on the medians of two independent variables. A forest plot based on median-divided group analysis was generated using the *forestploter* R package (v1.1.1). Survival differences between groups were visualized (Kaplan–Meier curves) using the *ggsurvfit* R package (v0.3.0); all indicated survival *p* values are from Cox proportional hazards models.

Multivariable Cox regression analysis (PBMC dataset) was performed using the adjusted model approach (the 10% rule for confounding^39^ with variables that were significant for univariable analysis, adjusting for relevant covariates from patient characteristics – considering those that had an univariable *p* ≤ 0.1—and including those that produced a > 10% change in the coefficient.

Linear Pearson correlations were computed on square root-transformed variables, and visualized either as circle correlograms or as bivariate scatter plot matrices, using the *ggcorrplot* (v0.1.4) and *psych* (v2.3.3) R packages. *p* values in this explorative analysis were not corrected for multiple testing. Correlations were visualized with hierarchical clustering using the complete linkage method. The scatterplot matrices show each available data point except for 1 outlier in both the IHC dataset and the mIF dataset, which were manually removed. *p* < .05 was considered statistically significant.

### Data availability

The data generated in this study, with anonymized patient IDs, are available upon reasonable request from the corresponding author.

## Results

PBMC immune profile analysis was performed on 67 blood samples from patients (Figure 1a). Within the baseline patient characteristics, age, primary tumor sidedness, differentiation grade, primary tumor *in situ*, number of metastases, number of organs involved in metastatic disease, and LDH status were associated with OS, and a number of these to progression-free survival (PFS) (Table **S**1). Liver, lung, lymph nodes, and the peritoneum were the most common metastatic sites, but no association with outcome was apparent for any specific organ in this cohort.

### Circulating immune cell subsets and survival

**Figure 2.**
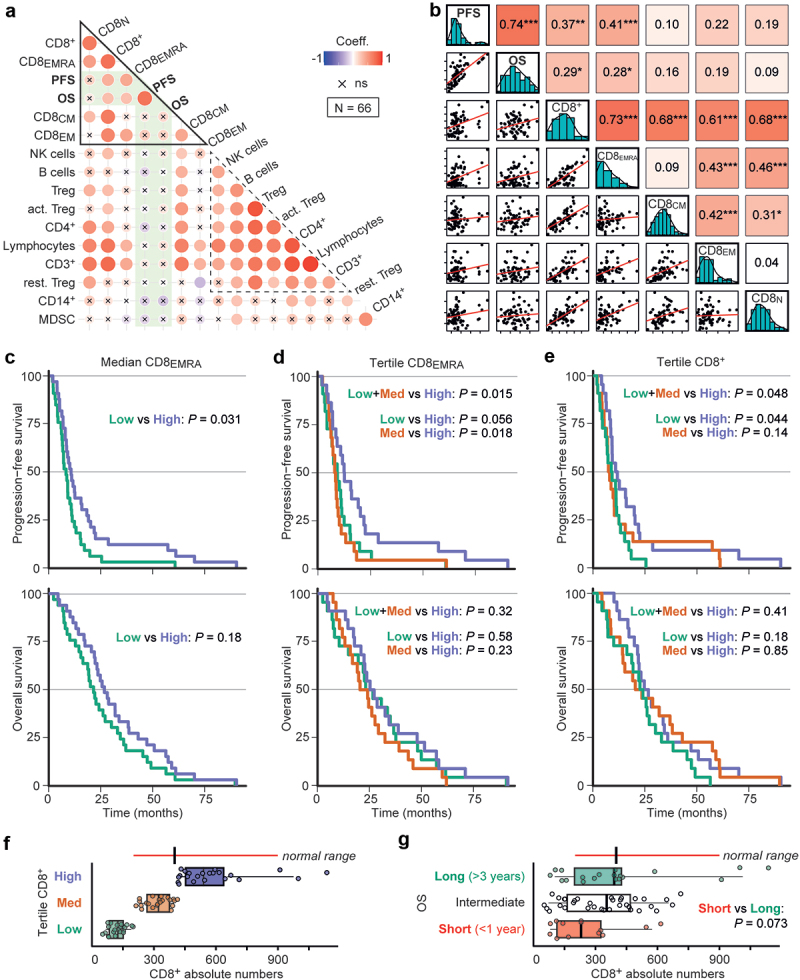
Analysis of circulating immune cell numbers in relation to patient survival.

To explore whether the local or systemic immune status correlated with survival, we first analyzed the systemic immune composition in the PBMC fraction of patient blood samples by flow cytometry (Figure S1). Absolute numbers of various immune cell populations across 67 patients were analyzed in a Pearson correlation matrix, together with OS and PFS. Cross-comparison of these variables followed by hierarchical clustering yielded 2 clusters (Figure 2a, triangles grouping red circles): CD8+ T cell variables co-clustering with survival, and the remaining lymphocytes. Positive correlations with survival were statistically significant for the total number of CD8+ T cells, as well as for CD8+ terminally differentiated effector (CD8_EMRA_) T cells (Figure 2b, shown as dot plots). Univariable Cox regression also indicated an association with OS and PFS for these variables (Table S2).

(a) Correlogram depicting linear Pearson correlations between absolute PBMC numbers, combined with PFS and OS (in months). Circle size and color indicate the correlation coefficient; an ‘x’ indicates a correlation that is not statistically significant (ns). Hierarchically clustering indicates two correlation clusters in the dataset, highlighted as triangles. (b) Pearson scatter plot matrix of the variables highlighted by the top triangle in (a), showing on the diagonal histograms and density of the selected variables. Bivariate scatter plots are shown below (x-axes are defined by column, y-axes by row), and the correlation coefficients. *P < .05; **P < .01; ***P < .001. c–e) Kaplan–Meier survival curves for the median-divided number of CD8_EMRA_ cells (c), or divided by tertiles for CD8_EMRA_ (d) or for CD8+ T cells (e). (f–g) CD8+ T cells per μl blood within the PBMC cohort, divided as in (e), or separated into short/intermediate/long survival. Individual data points and boxplots; both whiskers and normal-line depict the 5^th^–95^th^ percentile range. Comparison by an unequal-variances two-sided T-test.

Furthermore, dividing the patients using the median CD8_EMRA_ value resulted in median PFS (mPFS) of 10.8 *vs* 8.4 months (Figure 2c, Table 1A). Interestingly, subgroup survival analysis indicated that the ability for median-divided CD8_EMRA_ numbers to separate PFS only applied in patients with *RAS/BRAF* wildtype or left-sided CRCs (Figure S2). Survival separation was improved with a tertile CD8_EMRA_ separator: the high group had an improved mPFS compared to low+medium combined (12.5 *vs* 8.5 months; Figure 2d). Similar outcomes were found for tertiles in CD8+ T cells (high *vs* low+medium, 11.3 *vs* 8.3 months mPFS; Figure 2e). Comparing the latter with healthy adult reference values,^40^ the lower third of patients clearly had CD8+ T cell numbers below the normal range (Figure 2f). Moreover, dividing the PBMC dataset into patients with short/intermediate/long survival showed that 11/13 (85%) of short survival patients had fewer CD8+ T cells than the medians of other patients or healthy adults (Figure 2g). Other poor-prognosis biomarkers for comparison: 5/13 (38%) had a right-sided tumor, and among 10 short-survival patients with known *RAS/BRAF* mutation status, 5 (50%) were *KRAS*-mutant and 3 (30%) *BRAF*-mutant.

**Table 1A.** Hazard ratios for selected variables and PFS.

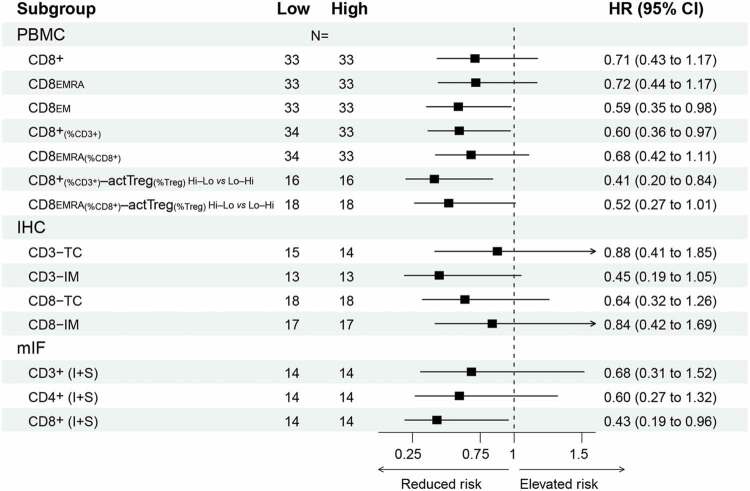

Median-divided data: higher *vs* lower.

**Figure 3.**
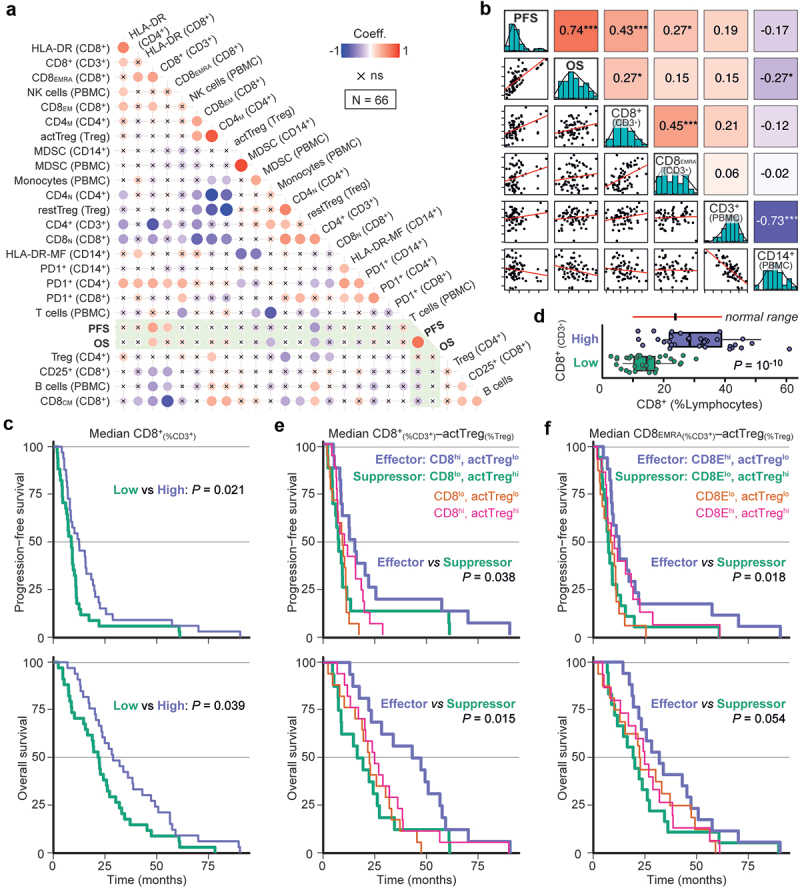
Analysis of circulating immune cell proportions in relation to patient survival.

Unlike for absolute values, there were no obvious correlation clusters when the Pearson cross-correlation analysis was repeated using relative PMBC measurements, e.g. percentages of parent population (Figure 3a, note the overall reduced circle sizes as well as the absence of a red group as seen in Figure 2a). Nevertheless, CD8+_(as %CD3+)_ and CD8_EMRA(%CD8+)_ T cells showed a positive correlation and association with PFS (Figure 3a,b and Table S3), and the former also with OS. Furthermore, median-divided high *vs* low CD8_EMRA(%CD8+)_ separated PFS, yielding 11.7 *vs* 7.7 months mPFS. High *vs* low CD8+_(%CD3+)_ had 12.4 *vs* 8.7 months mPFS and 28.8 *vs* 21.9 months mOS (HR for OS: 0.60, *p* = .039; Figure 3c and Table 1B). While this survival separation appeared again limited to patients with left-sided CRCs, survival separation by this variable seemed stronger in *KRAS*-mutant cancers (Figure **S**3a-d). In comparison with healthy adult reference values, our CD8+_(%CD3+)_-low patients had a much lower CD8+ population size (Figure 3d and S3E). These data indicate that reduced peripheral blood CD8+ numbers and effector phenotype are associated with shorter survival, possibly by poor immunological growth control of advanced CRC.

**Table 2B.** Hazard ratios for selected variables and OS.

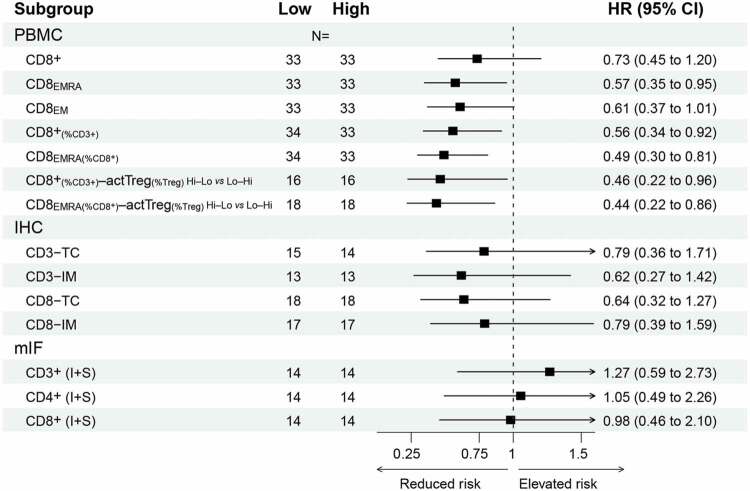

Median-divided data: higher vs lower.

(a) Pearson correlogram of proportional immune cell data (relative to the variable in brackets) and survival. (b) Pearson scatter plot matrix of the most relevant variables. *P < .05; **P < .01; ***P < .001. (c) Kaplan–Meier survival curves for median-divided CD8+_(%CD3+)_ T cells. (d) CD8+ T cell/lymphocyte percentage for the groups in (c), compared to normal range in healthy adults (10–39%). Individual data points and boxplots; both whiskers and normal-line depict the 5^th^–95^th^ percentile range. Comparison by an unequal-variances two-sided T-test. (e-f) Kaplan–Meier survival curves for combined assessments.

In line with a wider definition of a systemic effector phenotype, we analyzed the association with survival for different concurrences of either CD8+_(%CD3+)_ or CD8_EMRA(%CD8+)_ T cells with active Treg_(%Treg)_ cells (Table S3), similar to a high CD8:Treg ratio.^27,31^ For CD8+_(%CD3+)_–active Treg_(%Treg)_, we observed a difference in PFS between an effector (high – low) *vs* suppressor (low – high) phenotype; 13.9 *vs* 7.3 months mPFS and 45.1 *vs* 17.9 months mOS (HR for OS: 0.41, *p* = .015; Figure 3e). Especially for OS, the combined CD8+_(%CD3+)_—active Treg_(%Treg)_ assessment separated survival better than CD8+_(%CD3+)_ T cell status alone. Similarly, CD8_EMRA(%CD8+)_—active Treg_(%Treg)_ also separated survival: 12.5 *vs* 7.3 months mPFS, and 32.9 *vs* 19.7 months mOS (Figure 3f and Table 1B).

In multivariable Cox regression analysis for PBMC variables and baseline characteristics, the associations between CD8+ and CD8_EMRA_ T cells and OS/PFS retained statistical significance after adjusting for potential confounders (Table S4a,c). This indicates that high CD8+ T cell numbers in blood are independently associated with better survival. Additionally, the percentage of CD4+ T cells was independently associated with PFS (Table S4d).

### Primary tumour immune infiltration

**Figure 4.**
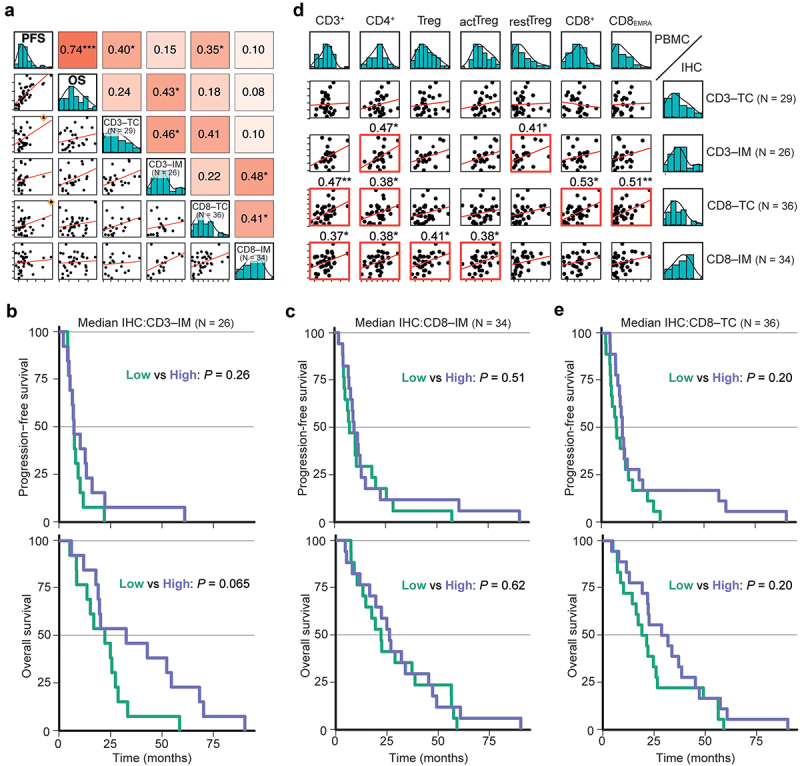
IHC analysis of primary CRC T cell infiltration in relation to patient survival.

We next investigated whether we could connect primary tumor T cell infiltration to survival in our cohort. Therefore, CD3+ and CD8+ T cell infiltration in both tumor center (TC) and invasive margin (IM) were assessed by immunohistochemistry. Unfortunately, due to uneven sample quality, the number of complete cases (with all four values, *N* = 19/45) was low. Nevertheless, a correlation between CD3–IM and OS, as well as correlations of both CD3–TC and CD8–TC with PFS were found, although the latter two are likely explained by outliers (Figure 4a, diamonds). Cox regression analyses indicated an association for CD3–TC with PFS, and for CD3-IM with OS (Table **S**5). By median-based division, only CD3-IM was able to separate OS somewhat (mOS 28.2 *vs* 19.3 months; Figure 4b,c and Table 1B).

a) Pearson scatter plot matrix of OS/PFS and T cell counts in either the tumor center (TC) or invasive margin (IM). Orange diamonds indicate outliers. b–c) Kaplan–Meier survival curves for the median-divided number of CD3+ (b) and CD8+ T cells (c) in IM. d) Pearson scatter plot matrix comparison between selected PBMC–IHC variables. Statistically significant correlations are indicated by a red outline and asterisks behind correlation coefficients. e) Kaplan–Meier survival curves for the median-divided number of CD8+ T cells in TC. *P < .05; **P < .01; ***P < .001.

Using a pairwise comparison of the IHC data with key PBMC variables, a correlation between high tumoral CD8–TC values and elevated circulating CD8+ and CD8_EMRA_ T cells was observed (Figure 4d). CD8–TC high *vs* low gave of 10.2 *vs* 7.3 months mPFS, and 30.4 *vs* 20.4 months mOS (Figure 4e and Table 1B). Therefore, despite interesting correlations between CD8+ T cells in primary tumors and in blood, our sample size was likely too small to obtain associations with survival.

### Liver metastasis lymphocyte infiltration for long vs short survivors

**Figure 5.**
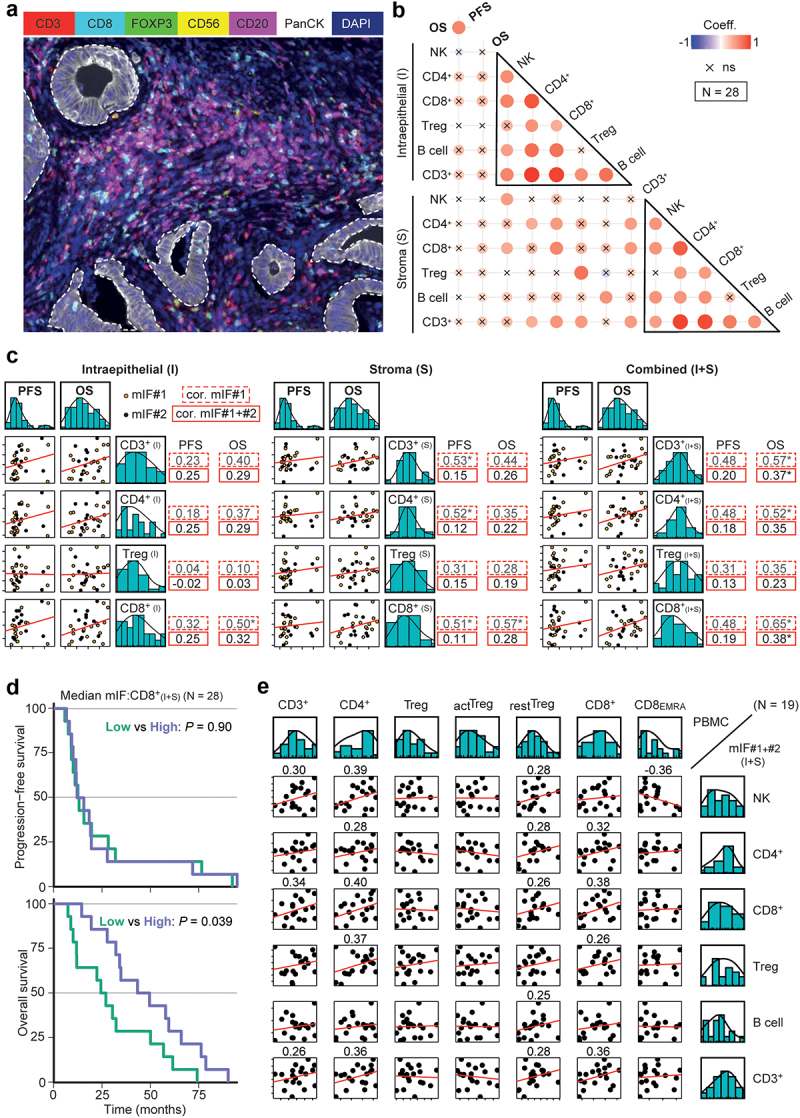
mIF analysis of liver metastasis lymphocyte infiltration linked to survival.

Finally, we performed multiplex immunofluorescence (mIF) on liver metastases to inspect the association of immunological variables with survival in advanced disease. First, an exploratory group of patients (mIF#1) with either short (*N* = 5) or long survival (*N* = 11) was analyzed – anticipating that this OS contrast might afford the best chance to uncover a potential link between immune features and metastatic growth control. Later, a second group (mIF#2, *N* = 12) was added to increase the overlap with the PBMC cohort, resulting in an OS distribution similar to the PMBC population (Figure 1b). The mIF panel included markers for T cells as well as for NK and B cells. With an additional tumor-identifying marker, cellular densities were assessed both inside glandular epithelial tumor beds (Intraepithelial) and in the surrounding tumor Stroma (Figure 5a). Correlations of CD8+ T cell density (I or S) in mIF#1 indicated an association with OS but were reduced in mIF#1+#2 and remained statistically significant only for total tumor (I+S) analyses (Figure 5b,c). In Cox regression analyses, both CD3+ and CD4+ T cell densities (I+S) associated with OS (Table **S**6). No such association was observed for subcompartments or for PFS.

(a) Liver metastasis section after mIF staining, showing epithelial tumor fields (dotted regions). (b) Linear Pearson correlations between CRLM immune cell densities and survival clustered by region. (c) Pearson scatter plot matrix of survival and T cell densities, by region. Datasets: mIF#1: orange/dotted-line boxes, #2: black dots; correlation lines and coefficients (solid boxes) are shown for #1+#2. *P < .05. (d) Kaplan–Meier survival curves for median-divided CD8+ T cell density (I+S). (e) Pearson scatter plot matrix of selected PBMC – mIF_(I+S)_ variables. Correlation coefficients are indicated unless between −0.25 and 0.25.

Furthermore, OS for these patients was associated with median-divided CD8+ T cell density in liver metastases (I+S), 46.1 *vs* 25.5 months mOS (HR 0.43, *p* = .039; Figure 5d and Table 1B). Indeed, all 5 short-survival patients were in the low group. Correlations between metastatic CD8+ T cell densities and PBMC CD8+ T cell numbers were positive but weak (*N* = 19, Figure 5e). This suggests that, while the convergence between the two techniques/tissue types is modest, the underlying biology may yet be conserved.

## Discussion

This exploratory study focused on a cohort of patients with advanced, multi-organ mCRC and indicates an association between elevated CD8+ T cell presence and improved survival. Notably, higher-than-median CD8+ T cell infiltration in liver metastasis associated with OS, as did a higher PBMC fraction of CD8+ T cells, and a CD8+_(%CD3+)_–active Treg_(%Treg)_ cell combined effector phenotype. Of particular interest was the contrast between patients who had a very short survival and those living longer. Our data suggest that low levels of both circulating and metastasis-infiltrating CD8+ T cell numbers may have prognostic value for short OS, potentially outperforming currently available biomarkers. Although metastatic location can have prognostic value,^41^ we did not find such an association here.

Whereas many studies have found similar associations between cancer immunity and survival in various cancer types,^42–45^ including in patients with early-stage CRC or resectable, single-organ mCRC treated with curative intent,^15–22,27–31^ this study is the first to assess more advanced (multi-organ metastatic) disease where treatment outcome is limited. This unique cohort was expected to present a lower immune status, compared to more limited disease. Indeed, the presence of multiple metastases was previously linked to a low immunoscore,^18^ representing poor T cell infiltration within metastatic lesions. In fact, only a modest survival difference was observed among their patients with many metastases when dividing over immunoscore.^18^ The apparent difference with our findings may be related to their low number of patients with multiple metastases.

The main limitation of the present exploratory, hypothesis-generating, study is the small size of the IHC/mIF datasets, as well as the limited overlap with the PBMC dataset. Furthermore, reducing measurements into two (or three) groups, divided by the median or tertiles, further reduces statistical power. For this reason, most of our univariable analyses were performed on continuous variables. Also, the PBMC dataset did not fully represent all already-validated prognostic biomarkers, such as *BRAF* mutation, which is likely a result of the inclusion criteria of the clinical trial. Future research should be sufficiently representative and powered to generate clinically relevant cutoffs for patient stratifications or treatment decision-making. Regarding the need for a better understanding of underlying biology, our study neither included a detailed focus on unconventional T cells (including NKT and γδ T cells,^46^ nor on suppressive/immature myeloid cells including neutrophils.^47,48^ Assessments with a wider scope will undoubtedly offer information on immunosuppressive mechanisms and may point to therapeutic opportunities.

## Conclusion and future outlook

If validated in a larger population, our results may help identify patients with absent or low cancer immunity that are likely to have a very short survival, despite systemic therapy. Once prognosticated, it can be considered to withhold systemic therapy in these patients to spare them unnecessary treatment-related toxicity. Moreover, this work supports further exploration of therapeutic strategies that could either boost adaptive immune responses that appear to characterize the relatively long survivors – potentially linking to better disease control – or elicit such responses where they seem absent. We foresee that such (combinatory) treatments will become available for patients with mCRC^12^ and several clinical trials in this area are ongoing.^49^ In earlier stages of CRC, immune therapy has shown potential even in pMMR/MSS tumors.^10^ Thus, our data may warrant the extension of immuno-oncology for mCRC to more advanced disease.

## Supplementary Material

Bakkerus_Figures_r1-Fig.S1.tif

Bakkerus_Figures_r1-Fig.S3.tif

Bakkerus_Figures_r1-Fig.S2.tif

## References

[1] Sung H, Ferlay J, Siegel RL, Laversanne M, Soerjomataram I, Jemal A, Bray F. Global cancer statistics 2020: GLOBOCAN estimates of incidence and mortality worldwide for 36 cancers in 185 countries. CA Cancer J Clin. 2021;71(3):209–12. doi:10.3322/caac.21660. 33538338

[2] Xie Y-H, Chen Y-X, Fang J-Y. Comprehensive review of targeted therapy for colorectal cancer. Signal Transduct Target Ther. 2020;5(1):22. doi:10.1038/s41392-020-0116-z. 32296018 PMC7082344

[3] Ciardiello F, Ciardiello D, Martini G, Napolitano S, Tabernero J, Cervantes A. Clinical management of metastatic colorectal cancer in the era of precision medicine. CA Cancer J Clin. 2022;72(4):372–401. doi:10.3322/caac.21728. 35472088

[4] Tauriello DVF, Calon A, Lonardo E, Batlle E. Determinants of metastatic competency in colorectal cancer. Mol Oncol. 2017;11(1):97–119. doi:10.1002/1878-0261.12018. 28085225 PMC5423222

[5] Zhou H, Liu Z, Wang Y, Wen X, Amador EH, Yuan L, Ran X, Xiong L, Ran Y, Chen W. et al. Colorectal liver metastasis: molecular mechanism and interventional therapy. Sig Transduct Target Ther. 2022;7(17):1–25. doi:10.1038/s41392-022-00922-2. PMC8897452 35246503

[6] Pagès F, Mlecnik B, Marliot F, Bindea G, Ou F-S, Bifulco C, Lugli A, Zlobec I, Rau TT, Berger MD. et al. International validation of the consensus Immunoscore for the classification of colon cancer: a prognostic and accuracy study. Lancet. 2018;391(10135):2128–2139. doi:10.1016/S0140-6736(18)30789-X. 29754777

[7] Bruni D, Angell HK, Galon J. The immune contexture and Immunoscore in cancer prognosis and therapeutic efficacy. Nat Rev Cancer. 2020;20(11):662–680. doi:10.1038/s41568-020-0285-7. 32753728

[8] Mellman I, Chen DS, Powles T, Turley SJ. The cancer-immunity cycle: Indication, genotype, and immunotype. Immunity. 2023;56(10):2188–2205. doi:10.1016/j.immuni.2023.09.011. 37820582

[9] Angelova M, Mlecnik B, Vasaturo A, Bindea G, Fredriksen T, Lafontaine L, Buttard B, Morgand E, Bruni D, Jouret-Mourin A. et al. Evolution of metastases in space and time under immune selection. Cell. 2018;175(3):751–765.e16. doi:10.1016/j.cell.2018.09.018. 30318143

[10] Chalabi M, Fanchi LF, Dijkstra KK, Van den Berg JG, Aalbers AG, Sikorska K, Lopez-Yurda M, Grootscholten C, Beets GL, Snaebjornsson P. et al. Neoadjuvant immunotherapy leads to pathological responses in MMR-proficient and MMR-deficient early-stage colon cancers. Nat Med. 2020;26(4):566–576. doi:10.1038/s41591-020-0805-8. 32251400

[11] Tauriello DVF, Palomo-Ponce S, Stork D, Berenguer-Llergo A, Badia-Ramentol J, Iglesias M, Sevillano M, Ibiza S, Cañellas A, Hernando-Momblona X. et al. TGFβ drives immune evasion in genetically reconstituted colon cancer metastasis. Nature. 2018;554(7693):538–543. doi:10.1038/nature25492. 29443964

[12] Janssen E, Subtil B, de la Jara Ortiz F, Verheul HMW, Tauriello DVF. Combinatorial immunotherapies for metastatic colorectal cancer. Cancers Basel. 2020;12(7):1875. doi:10.3390/cancers12071875. 32664619 PMC7408881

[13] Le DT, Durham JN, Smith KN, Wang H, Bartlett BR, Aulakh LK, Lu S, Kemberling H, Wilt C, Luber BS. et al. Mismatch repair deficiency predicts response of solid tumors to PD-1 blockade. Science. 2017;357(6349):409–413. doi:10.1126/science.aan6733. 28596308 PMC5576142

[14] Halama N, Michel S, Kloor M, Zoernig I, Benner A, Spille A, Pommerencke T, von Knebel DM, Folprecht G, Luber B. et al. Localization and density of immune cells in the invasive margin of human colorectal cancer liver metastases are prognostic for response to chemotherapy. Cancer Res. 2011;71(17):5670–5677. doi:10.1158/0008-5472.CAN-11-0268. 21846824

[15] Tanis E, Julié C, Emile J-F, Mauer M, Nordlinger B, Aust D, Roth A, Lutz MP, Gruenberger T, Wrba F. et al. Prognostic impact of immune response in resectable colorectal liver metastases treated by surgery alone or surgery with perioperative FOLFOX in the randomised EORTC study 40983. Eur J Cancer. 2015;51(17):2708–2717. doi:10.1016/j.ejca.2015.08.014. 26342674

[16] Kwak Y, Koh J, Kim D-W, Kang S-B, Kim WH, Lee HS. Immunoscore encompassing CD3+ and CD8+ T cell densities in distant metastasis is a robust prognostic marker for advanced colorectal cancer. Oncotarget. 2016;7(49):81778–81790. doi:10.18632/oncotarget.13207. 27835889 PMC5348429

[17] Brunner SM, Kesselring R, Rubner C, Martin M, Jeiter T, Boerner T, Ruemmele P, Schlitt HJ, Fichtner-Feigl S. Prognosis according to histochemical analysis of liver metastases removed at liver resection. Br J Surg. 2014;101(13):1681–1691. doi:10.1002/bjs.9627. 25331841

[18] Van den Eynde M, Mlecnik B, Bindea G, Fredriksen T, Church SE, Lafontaine L, Haicheur N, Marliot F, Angelova M, Vasaturo A. et al. The link between the multiverse of immune microenvironments in metastases and the survival of colorectal cancer patients. Cancer Cell. 2018;34(6):1012–1026.e3. doi:10.1016/j.ccell.2018.11.003. 30537506

[19] Mlecnik B, Van den Eynde M, Bindea G, Church SE, Vasaturo A, Fredriksen T, Lafontaine L, Haicheur N, Marliot F, Debetancourt D. et al. Comprehensive intrametastatic immune quantification and major impact of immunoscore on survival. JNCI: J Nat Cancer Inst. 2018;110(1):97–108. doi:10.1093/jnci/djx123. 28922789

[20] Kamal Y, Schmit SL, Frost HR, Amos CI. The tumor microenvironment of colorectal cancer metastases: opportunities in cancer immunotherapy. Immunotherapy. 2020;12(14):1083–1100. doi:10.2217/imt-2020-0026. 32787587 PMC8411393

[21] Höppener DJ, Nierop PMH, Hof J, Sideras K, Zhou G, Visser L, Gouw ASH, de Jong KP, Sprengers D, Kwekkeboom J. et al. Enrichment of the tumour immune microenvironment in patients with desmoplastic colorectal liver metastasis. Br J Cancer. 2020;123(2):196–206. doi:10.1038/s41416-020-0881-z. 32418992 PMC7374625

[22] Liu Y, Zhang Q, Xing B, Luo N, Gao R, Yu K, Hu X, Bu Z, Peng J, Ren X. et al. Immune phenotypic linkage between colorectal cancer and liver metastasis. Cancer Cell. 2022;40(4):424–437.e5. doi:10.1016/j.ccell.2022.02.013. 35303421

[23] Wargo JA, Reddy SM, Reuben A, Sharma P. Monitoring immune responses in the tumor microenvironment. Curr Opin Immunol. 2016;41:23–31. doi:10.1016/j.coi.2016.05.006. 27240055 PMC5257261

[24] Stroncek DF, Butterfield LH, Cannarile MA, Dhodapkar MV, Greten TF, Grivel JC, Kaufman DR, Kong HH, Korangy F, Lee PP. et al. Systematic evaluation of immune regulation and modulation. J Immunother Cancer. 2017;5(1):21. doi:10.1186/s40425-017-0223-8. 28331613 PMC5359947

[25] Allen BM, Hiam KJ, Burnett CE, Venida A, DeBarge R, Tenvooren I, Marquez DM, Cho NW, Carmi Y, Spitzer MH. Systemic dysfunction and plasticity of the immune macroenvironment in cancer models. Nat Med. 2020;26(7):1125–1134. doi:10.1038/s41591-020-0892-6. 32451499 PMC7384250

[26] Hiam-Galvez KJ, Allen BM, Spitzer MH. Systemic immunity in cancer. Nat Rev Cancer. 2021;21(6):345–359. doi:10.1038/s41568-021-00347-z. 33837297 PMC8034277

[27] Roselli M, Formica V, Cereda V, Jochems C, Richards J, Grenga I, Orlandi A, Ferroni P, Guadagni F, Schlom J. et al. The association of clinical outcome and peripheral T-cell subsets in metastatic colorectal cancer patients receiving first-line FOLFIRI plus bevacizumab therapy. Oncoimmunology. 2016;5(7):e1188243. doi:10.1080/2162402X.2016.1188243. 27622042 PMC5006891

[28] Tada K, Kitano S, Shoji H, Nishimura T, Shimada Y, Nagashima K, Aoki K, Hiraoka N, Honma Y, Iwasa S. et al. Pretreatment immune status correlates with progression-free survival in chemotherapy-treated metastatic colorectal cancer patients. Cancer Immunol Res. 2016;4(7):592–599. doi:10.1158/2326-6066.CIR-15-0298. 27197061

[29] Shibutani M, Maeda K, Nagahara H, Fukuoka T, Matsutani S, Kashiwagi S, Tanaka H, Hirakawa K, Ohira M. A comparison of the local immune status between the primary and metastatic tumor in colorectal cancer: a retrospective study. BMC Cancer. 2018;18(1):371. doi:10.1186/s12885-018-4276-y. 29614981 PMC5883878

[30] Krijgsman D, de Vries NL, Skovbo A, Andersen MN, Swets M, Bastiaannet E, Vahrmeijer AL, van de Velde CJH, Heemskerk MHM, Hokland M. et al. Characterization of circulating T-, NK-, and NKT cell subsets in patients with colorectal cancer: the peripheral blood immune cell profile. Cancer Immunol Immun. 2019;68(6):1011–1024. doi:10.1007/s00262-019-02343-7. PMC6529387 31053876

[31] Bencsikova B, Budinska E, Selingerova I, Pilatova K, Fedorova L, Greplova K, Nenutil R, Valik D, Obermannova R, Sheard MA. et al. Circulating T cell subsets are associated with clinical outcome of anti-VEGF-based 1st-line treatment of metastatic colorectal cancer patients: a prospective study with focus on primary tumor sidedness. BMC Cancer. 2019;19(1):687. doi:10.1186/s12885-019-5909-5. 31307428 PMC6631500

[32] Schnell A, Schmidl C, Herr W, Siska PJ. The peripheral and intratumoral immune cell landscape in cancer patients: a proxy for tumor biology and a tool for outcome prediction. Biomedicines. 2018;6(1):25. doi:10.3390/biomedicines6010025. 29495308 PMC5874682

[33] Lutke Schipholt IJ, Scholten-Peeters GGM, Koop MA, Bonnet P, Bontkes HJ, Coppieters MW. Systemic neuroimmune responses in people with non-specific neck pain and cervical radiculopathy, and associations with clinical, psychological, and lifestyle factors. Front Mol Neurosci. 2022;15:1003821. doi:10.3389/fnmol.2022.1003821. 36311017 PMC9608367

[34] Huijts CM, Lougheed SM, Bodalal Z, van Herpen CM, Hamberg P, Tascilar M, Haanen JB, Verheul HM, de Gruijl TD, van der Vliet HJ. et al. The effect of everolimus and low-dose cyclophosphamide on immune cell subsets in patients with metastatic renal cell carcinoma: results from a phase I clinical trial. Cancer Immunol Immunother. 2019;68(3):503. doi:10.1007/s00262-018-2288-8. 30652208 PMC6426984

[35] Berry S, Giraldo NA, Green BF, Cottrell TR, Stein JE, Engle EL, Xu H, Ogurtsova A, Roberts C, Wang D. et al. Analysis of multispectral imaging with the AstroPath platform informs efficacy of PD-1 blockade. Science. 2021;372(6547). doi:10.1126/science.aba2609. PMC8709533 34112666

[36] Swiderska-Chadaj Z, Pinckaers H, van Rijthoven M, Balkenhol M, Melnikova M, Geessink O, Manson Q, Sherman M, Polonia A, Parry J. et al. Learning to detect lymphocytes in immunohistochemistry with deep learning. Med Image Anal. 2019;58:101547. doi:10.1016/j.media.2019.101547. 31476576

[37] Gorris MAJ, Halilovic A, Rabold K, van Duffelen A, Wickramasinghe IN, Verweij D, Wortel IMN, Textor JC, de Vries IJM, Figdor CG. et al. Eight-color multiplex immunohistochemistry for simultaneous detection of multiple immune checkpoint molecules within the tumor microenvironment. J Immunol. 2018;200(1):347–354. doi:10.4049/jimmunol.1701262. 29141863

[38] Sultan S, Gorris, MA, Martynova E, van der Woude, LL, Buytenhuijs F, van Wilpe, S, Verrijp K, Figdor, CG, de Vries, IJ, Textor J. ImmuNet: a segmentation-free machine learning pipeline for immune landscape phenotyping in tumors by muliplex imaging. bioRxiv. 2023;41. doi:10.1101/2021.10.22.464548. PMC11769680 39866377

[39] Twisk JWR. Applied mixed model analysis: a practical guide. Cambridge: Cambridge University Press; 2019.

[40] Comans-Bitter WM, de Groot R, van den Beemd R, Neijens HJ, Hop WCJ, Groeneveld K, Hooijkaas H, van Dongen JJM. Immunophenotyping of blood lymphocytes in childhood. Reference values for lymphocyte subpopulations. J Pediatr. 1997;130(3):388–393. doi:10.1016/S0022-3476(97)70200-2. 9063413

[41] Wang J, Li S, Liu Y, Zhang C, Li H, Lai B. Metastatic patterns and survival outcomes in patients with stage IV colon cancer: A population-based analysis. Cancer Med. 2020;9(1):361–373. doi:10.1002/cam4.2673. 31693304 PMC6943094

[42] Pernot S, Terme M, Radosevic-Robin N, Castan F, Badoual C, Marcheteau E, Penault-Llorca F, Bouche O, Bennouna J, Francois E. et al. Infiltrating and peripheral immune cell analysis in advanced gastric cancer according to the Lauren classification and its prognostic significance. Gastric Cancer. 2020;23(1):73–81. doi:10.1007/s10120-019-00983-3. 31267360

[43] Hu Z, Zhou J, Li Y, Luan Y, Li H, Jia B, Xie Z, Cheng B, Wu T. Peripheral immune signature resembles tumor microenvironment and predicts clinical outcomes in head and neck squamous cell carcinoma. Front Immunol. 2022;13:915207. doi:10.3389/fimmu.2022.915207. 36148222 PMC9486472

[44] Wu TD, Madireddi S, de Almeida PE, Banchereau R, Chen YJJ, Chitre AS, Chiang EY, Iftikhar H, O’Gorman WE, Au-Yeung A. et al. Peripheral T cell expansion predicts tumour infiltration and clinical response. Nature. 2020;579(7798):274–278. doi:10.1038/s41586-020-2056-8. 32103181

[45] Zhang W, Ling Y, Li Z, Peng X, Ren Y. Peripheral and tumor-infiltrating immune cells are correlated with patient outcomes in ovarian cancer. Cancer Med. 2023;12(8):10045–10061. doi:10.1002/cam4.5590. 36645174 PMC10166954

[46] de Vries NL, van de Haar J, Veninga V, Chalabi M, Ijsselsteijn ME, van der Ploeg M, van den Bulk J, Ruano D, van den Berg JG, Haanen JB. et al. γδ T cells are effectors of immunotherapy in cancers with HLA class I defects. Nature. 2023;613(7945):743–750. doi:10.1038/s41586-022-05593-1. 36631610 PMC9876799

[47] Mizuno R, Kawada K, Itatani Y, Ogawa R, Kiyasu Y, Sakai Y. The role of tumor-associated neutrophils in colorectal cancer. Int J Mol Sci. 2019;20(3):529. doi:10.3390/ijms20030529. 30691207 PMC6386937

[48] Sieminska I, Baran J. Myeloid-derived suppressor cells in colorectal cancer. Front Immunol. 2020;11:1526. doi:10.3389/fimmu.2020.01526. 32849517 PMC7426395

[49] Shu Y, Zheng S. The current status and prospect of immunotherapy in colorectal cancer. Clin Transl Oncol. 2024;26(1):39–51. doi:10.1007/s12094-023-03235-0. 37301804

