# Supplementary figures and images for: Exploring immune status in peripheral blood and tumor tissue in association with survival in patients with multi-organ metastatic colorectal cancer
Source: Oncoimmunology. 2024 Jun 10;13(1):2361971. doi: 10.1080/2162402X.2024.2361971 (PMC11168219; doi:10.1080/2162402X.2024.2361971)

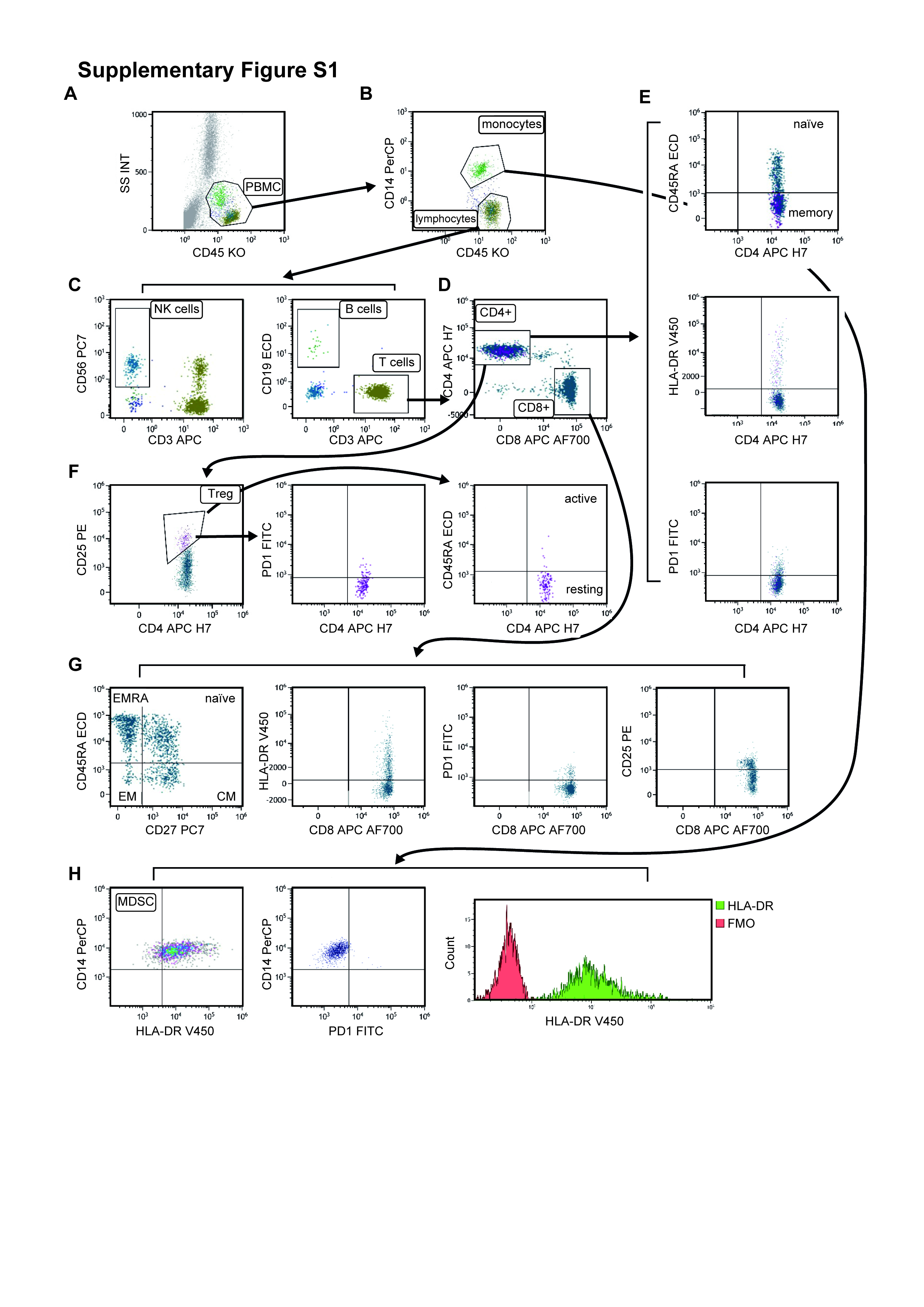

Supplement: Bakkerus_Figures_r1-Fig.S1.tif [file KONI_A_2361971_SM3858.tif]

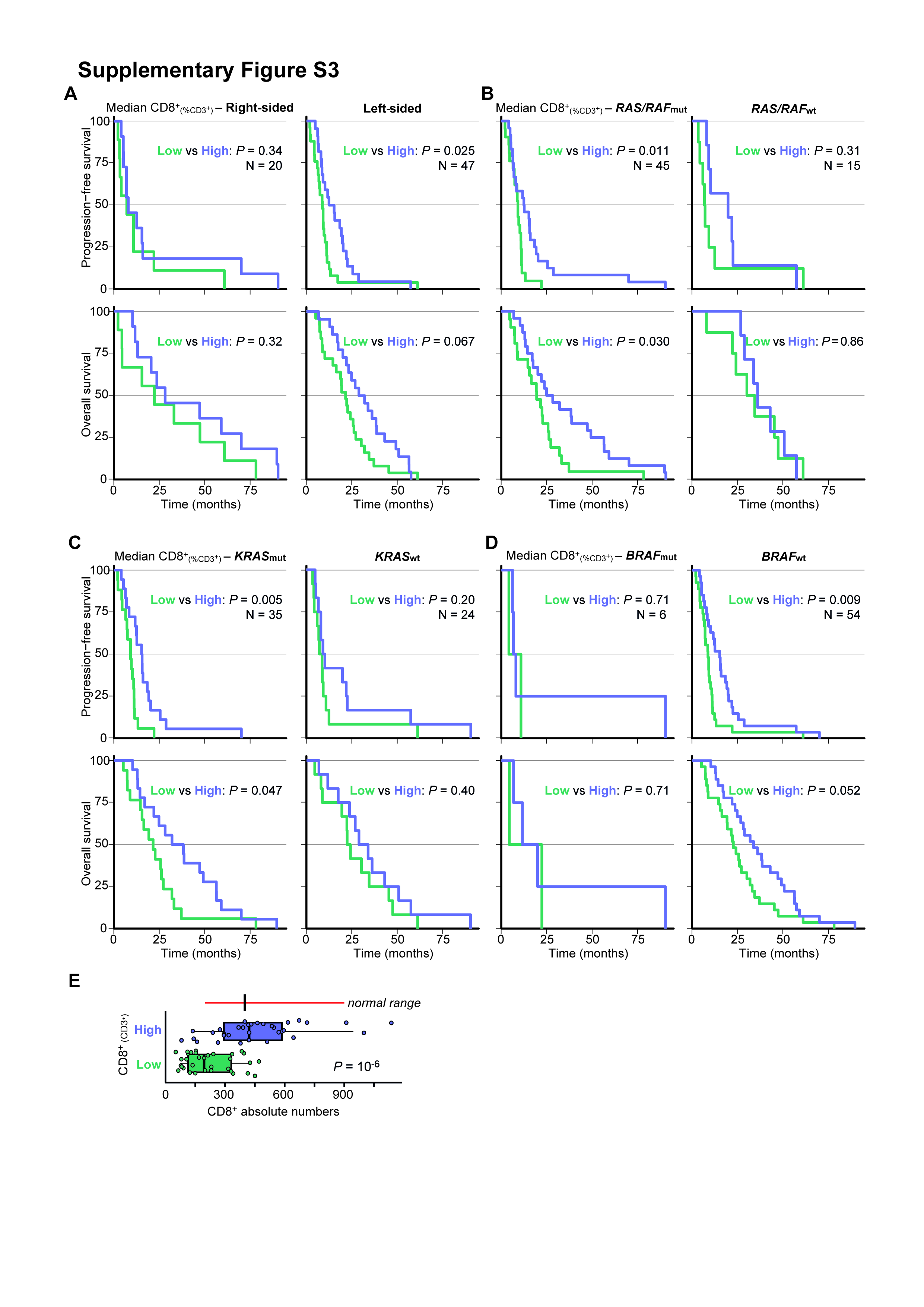

Supplement: Bakkerus_Figures_r1-Fig.S3.tif [file KONI_A_2361971_SM3857.tif]

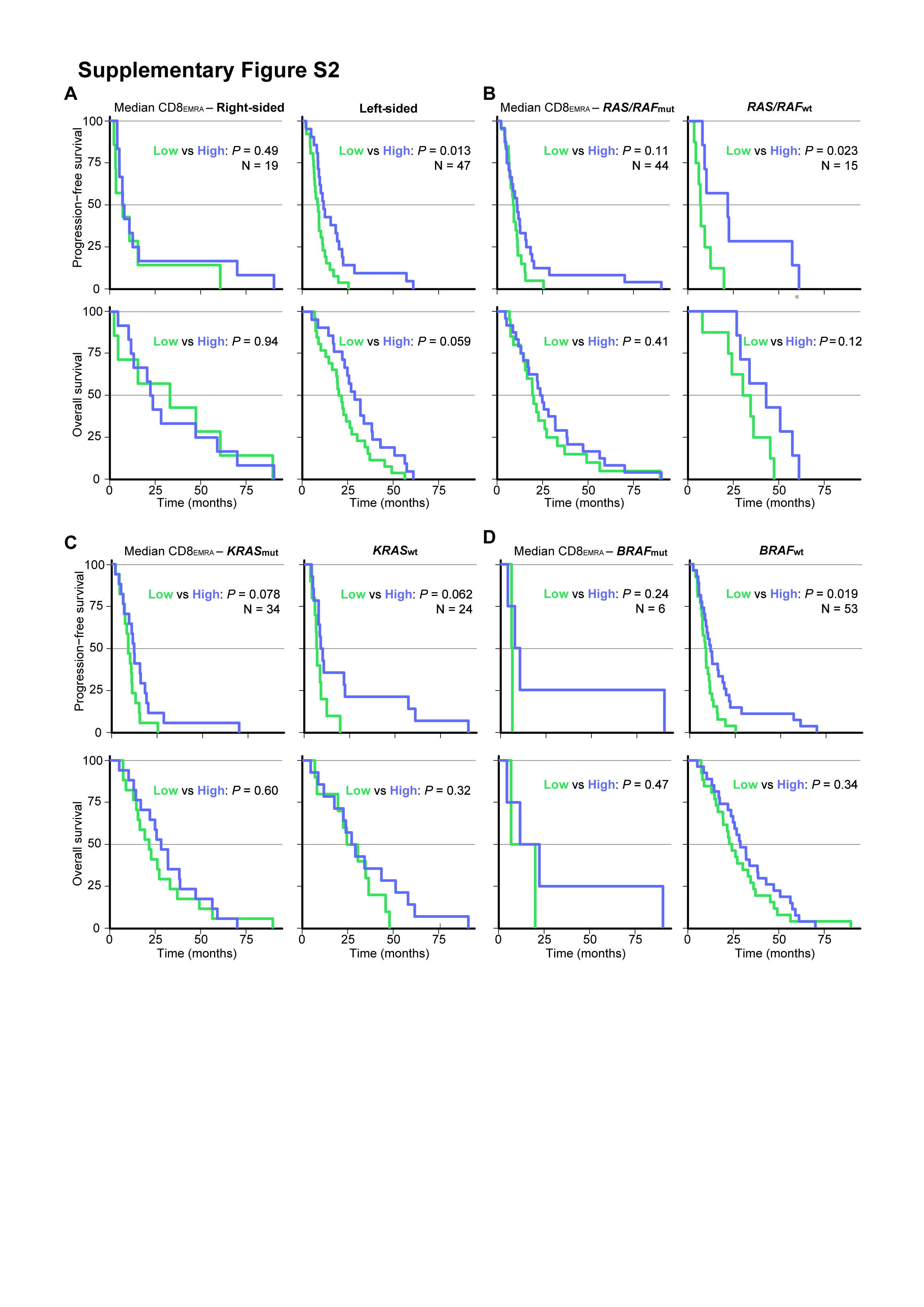

Supplement: Bakkerus_Figures_r1-Fig.S2.tif [file KONI_A_2361971_SM3856.tif]
